# Tumor Microenvironment-Induced Immunometabolic Reprogramming of Natural Killer Cells

**DOI:** 10.3389/fimmu.2018.02517

**Published:** 2018-11-08

**Authors:** Andrea M. Chambers, Kyle B. Lupo, Sandro Matosevic

**Affiliations:** ^1^Department of Industrial and Physical Pharmacy, Purdue University, West Lafayette, IN, United States; ^2^Center for Cancer Research, Purdue University, West Lafayette, IN, United States

**Keywords:** immunometabolism, natural killer cells, immunotherapy, tumor microenvironment, adenosine

## Abstract

Energy metabolism is key to the promotion of tumor growth, development, and metastasis. At the same time, cellular metabolism also mediates immune cell survival, proliferation and cytotoxic responses within the tumor microenvironment. The ability of natural killer cells to eradicate tumors relies on their ability to functionally persist for the duration of their anti-tumor effector activity. However, a tumor's altered metabolic requirements lead to compromised functional responses of cytokine-activated natural killer cells, which result in decreased effectiveness of adoptive cell-based immunotherapies. Tumors exert these immunosuppressive effects through a number of mechanisms, a key driver of which is hypoxia. Hypoxia also fuels the generation of adenosine from the cancer-associated ectoenzymes CD39 and CD73. Adenosine's immunosuppression manifests in decreased proliferation and impaired anti-tumor function, with adenosinergic signaling emerging as an immunometabolic checkpoint blockade target. Understanding such immunometabolic suppression is critical in directing the engineering of a new generation of natural killer cell-based immunotherapies that have the ability to more effectively target difficult-to-treat solid tumors.

## Introduction

Warburg metabolism, alongside glutamine turnover, is a hallmark of cancer, associated with elevated metabolism of glucose to produce ATP and sustain rapid cancer cell growth (1). Such metabolic reprogramming from glycolysis to oxidative phosphorylation results in the activation of a number of signaling pathways, upregulation of oncogenes and transcriptional factors, and inactivation of tumor suppressor genes. Similarly, natural killer (NK) cells undergo tumor-induced metabolic reprogramming, when they switch from a basal to an activated state. Such reprogramming leads to altered cytotoxic function, and can result in impaired effectiveness of immunotherapies relying on adoptively-transferred NK cells *in vivo*. The deregulated metabolism in the tumor microenvironment (TME) exerts an immunosuppressive effect on NK cells through a combination of mechanisms (Table 1). Some of the most representative features of an immunosuppressive TME are environmental heterogeneity to include deep hypoxic cores which lead to the generation of suppressive metabolites, such as adenosine, and the secretion of metabolic by-products. Though adoptive immunotherapy of both unmodified (9) and genetically-engineered NK cells (14) has been extensively demonstrated, the implication of immunometabolism is only starting to be investigated. Most of the insights into immunometabolic suppression of NK cell function currently originate from a limited number of focused studies. Until now, the interest has largely been on improving the cytotoxic activity of NK cells and enhancing target recognition.

**Table 1:** Sources of immunometabolic inhibition of NK cell functions.

**Source**	**Immunometabolic regulator**	**Effect on NK cells**	**References**
Cancer cells	LDHA-associated lactic acid production	Downregulation of NFAT; inhibition of effector functions	(2)
NK cells	HIF-1α	Downregulation of NKp46, NKp30, NKp44, and NKG2D, inactivation of mTOR	(3), (4)
Cancer cells (renal cancer)	HIF-2α	Upregulation of ITPR1 to regulate autophagy through calcium signaling	(5)
Irregular vascularization/Tumor microenvironment	Hypoxia	Generation of adenosine and reduction in NK cytotoxic function, Overexpression of HIF proteins (1α and 2α)	(6), (7), (8)
Cancer cells/Tumor microenvironment	Extracellular adenosine	Binding to A_2A_R receptor to inhibit proliferation, reduction of cytokine production, and reduction of cytotoxic function	(9), (10), (11)
Exogenous source	Rapamycin or Torin-1 (metabolic inhibitors)	Inhibition of the mTOR pathway	(12)
Exogenous source	Overstimulation with IL-15	Diminished cytolytic and inflammatory function	(13)

The use of natural killer cells in adoptive immunotherapy has traditionally relied on equipping these cells with synthetic machinery—such as genetically-engineered chimeric antigen receptors—to improve target recognition (14, 15). It is becoming increasingly apparent that next-generation immunotherapies, for difficult-to-treat solid tumors, will require the consideration of the emerging role of immunometabolism on immune function. Here, we discuss the current state-of-the-art of the field and the approaches aimed at targeting immunometabolic suppression to improve adoptive NK cell immunotherapy.

## Natural killer cell subsets

The function of NK cells is regulated by the interplay of a number of activating and inhibitory receptors. NK cells are considered to be the most well-known subset of innate lymphoid cells, which also includes tissue inducer cells and non-cytotoxic innate lymphoid cells (ILCs). Though both human and mouse innate lymphoid cells include conventional NK cells, non-cytotoxic innate lymphoid cell groups 1, 2, and 3 (ILC1, ILC2, and ILC3) as well as intraepithelial innate lymphoid cell group 1 (ieILC1), these cells differ in their expression of surface markers. For instance, murine ILCs do not express CD56 and express NK1.1 instead of NKp44 (16).

Phenotypically, human NK cells are defined by the expression of CD56 and the absence of CD3. NK cells are then additionally split into two main subsets based on the expression of CD56 and CD16: CD56^bright^CD16^±^ and CD56^dim^CD16^bright(+)^. These subsets differ in tissue distribution, and are dependent on specific homing properties and the cells' *in situ* maturation. CD56^dim^CD16^bright^ cell represent about 90% of all NK cells, and are predominant in peripheral blood. On the other hand, CD56^bright^CD16^±^, found mostly in lymphoid organs, can be subdivided into CD16^−^ (which represent about 30–50% of CD56^bright^ cells), and CD16^dim^ (50–70% of CD56^bright^) subsets. The less common CD56^dim^CD16^−^ and CD56^−^CD16^+^ cells have also been described, but the function of these cells is not well-known (17). Over 90% of peripheral blood NK cells are also killer immunologlobulin-like receptor (KIR)^+^.

Distribution and trafficking of NK cells in tissues has been extensively described (18). Tissue-resident NK cells express CD69, which blood-derived NK cells lack (19). They also differ in expression of chemokine receptors and adhesion molecules: Tissue resident NK cells tend to express CXCR6 and CCR5 and the integrins CD49a and CD103, while blood-derived NK cells express CXCR3, CXCR4, CCR7, CD62L (L-selectin), and lack CD49a (20).

Murine NK cells differ from human NK cells in a few notable aspects. While human NK cells express KIRs, mouse NK cells are characterized by expression of the C-type lectin-like family of receptors, Ly49s. Mouse NK cells, additionally, lack expression of CD56, which is a hallmark of human NK cells.

Murine NK cells are primarily defined based on their expression of CD27 and CD11b. In adult mice, CD11b^low^ cells are primarily found in the bone marrow, lymph nodes and the liver, while the CD11b^high^ subset is located in peripheral blood, the spleen and lungs. Among these, the CD11b^high^CD27^high^ subset is the most highly cytotoxic and expresses higher amounts of cytokines (21). Correlations have been made in terms of functionality between CD11b^low^CD27^high^ and CD11b^high^CD27^low^ NK cells in mice with CD56^bright^ and CD56^dim^ in humans, respectively (22). The intratumoral infiltration of these subsets also differs, with CD27^+^CD11b^+^ the prevalent subset found in fibrosarcoma (23). Mouse NK cells also express NK1.1, CD16 and CD122 and are regulated by different activating and inhibitory receptors (24).

## Immunometabolic cytokine activation of NK cells

Insights into the metabolism of natural killer cells mostly come from studies using murine cells, though a rapidly increasing body of work is contributing to our expanding knowledge of human NK cells. Glycolytic fueling in tumors reduces glucose availability to surrounding immune cells, leading to their metabolic reprogramming (25). In NK cells, regulation of metabolic response by up-regulation of glucose uptake and glycolysis is mediated by mTOR, specifically mTORC1 (26). mTORC1 activation requires sufficient intracellular nutrients and energy. mTOR is also essential for regulating the production of granzyme B and perforin, and can most potently be activated with high-concentrations of IL-15 during early infection, though other cytokines (IL-2, IL-12, IL-18) are also implicated (27). IL-15 activates mTORC1 via PI3K, PDPK1, and AKT (28). While NK cells do not exhibit increased glycolysis during short-term activation, extended stimulation with high-dose IL-15 over multiple days was shown to lead to up-regulation of metabolism, enhancing glycolysis (29). mTORC1 also enhances glycolysis by promoting transcription factor HIFα and mitochondrial biogenesis through PPARγ co-activator 1α (PGC1α) and yin and yang 1 (YY1) (30). Recently, Srebp, otherwise implicated in *de novo* lipid synthesis, has been shown to regulate functional responses and NK cell effector function, in supporting glycolysis and oxidative phosphorylation by the use of the citrate–malate shuttle, through its targets *Acly* and *Slc25a1* (31). High rates of glycolysis in tumors exert inhibitory effects on tumor-infiltrating NK cells also via cancer-associated lactate dehydrogenase-A (LDHA). LDHA fuels the conversion of excess pyruvate and NADH into lactate and NAD^+^, thus supporting tumor glycolysis. Brand et al. (2) recently reported that LDHA-associated lactic acid production leads to impaired NK cell activity through downregulation of nuclear factor of activated T cells (NFAT) in T and NK cells.

In response to diminishing glucose supplies, NK cells are thought to undergo metabolic reprogramming by foregoing IL-15 and mTOR dependency, and instead becoming driven by activating receptors (e.g., Ly49H in mice, KIR in humans) (32). However, the metabolic reprogramming of failed NK metabolism in a tumor setting has not been investigated in detail. This reprogramming is likely to be activation-dependent: cytokine-stimulated NK cells can produce IFN-γ independent of glycolysis or mitochondrial oxidative phosphorylation, while activating-receptor stimulated NK cells require oxidative phosphorylation (29).

Though scarce, evidence is also emerging that metabolic signatures also differ among human NK cell subsets, albeit from limited *in vitro* studies. In addition to greater activation of mTORC1, cytokine-activated CD56^bright^ cells are thought to have higher rates of glucose uptake compared with CD56^dim^ cells, associated with their higher expression of IFNγ (33). While CD56^dim^ cells are more cytotoxically active, they also have a lower biosynthetic burden and are likely to have lower metabolic requirements (33).

Because cytokines are a critical feature of adoptive immunotherapies with NK cells, understanding their activation-specific metabolic requirements to engage in anti-cancer cytotoxic functions is critical to the implementation of NK cells as viable immunotherapies.

## Hypoxia-induced metabolic reprogramming of NK cells

Oxygen availability is dependent on the metabolic requirements and functional status of each organ. During immunological responses in inflammatory and tumor microenvironments, NK cells operate at varying concentrations of oxygen, often reaching regions of severely low oxygen (hypoxia). Hypoxia is, as such, considered an adverse prognostic factor, particularly for solid tumors (34), and has been documented as being a feature of multiple pathologies (35). While oxygen concentrations in human tissues range from as low as 1.3% in bone marrow (36) to 13% in arterial blood (37), the existence of a hypoxic niche at sites of inflammation and in pathological environments has been evidenced by measured oxygen concentrations that are ~3–8 times lower than in corresponding physioxic tissues (38).

Tumor microenvironments are characterized by cycling hypoxia (39), throughout which they are exposed to bursts of varying concentrations of oxygen due to irregular vascularization and blood supply of tumor tissues. Both acute and chronic hypoxia result in DNA damage (40), replication arrest (41), radioresistance (42), epithelial to mesenchymal transition (43), angiogenesis and metastasis (44), and immune resistance (45).

Key NK cell responses to hypoxia are regulated by overexpression of HIF-1α and HIF-2α (6, 7), although inconclusive mechanisms have been proposed for their exact effect on NK cells. HIF-α can signal through and is stabilized via both oxygen-dependent and oxygen-independent mechanisms (46). These transcription factors direct responses of NK cells to low oxygen, influencing trophoblast lineage commitment and promoting development of the invasive trophoblast lineage during fetal development (47).

The overexpression of HIF-1α in hypoxic environments, which is partially dependent on mTOR signaling, leads to the downregulation of NK activating receptors NKp46, NKp30, NKp44, and NKG2D (3). Recent work has shown that deletion of HIF-1α in NK cells renders them hyporesponsive in both hypoxia and normoxia. Under long term hypoxia, tumor-associated NK cells from HIF-1α KO mice did not show a reduction of soluble vascular endothelial growth factor receptor 1 (sVEGFR1) expression, likely compensated by HIF-2α. These cells were also found to be less present in hypoxic zones, suggesting HIF-1α has an effect on intratumoral infiltration of NK cells and, collectively, increasing the bioavailability of VEGF (48). Others have also hypothesized that during long-term hypoxia, the HIF-1α protein accumulates due to the inactivation of its degradation pathway. This buildup of HIF-1α enforces a negative feedback loop to inactivate mTOR and cause diminished cellular translation and deleterious downstream effects (4).

Additionally, hypoxia was shown to cause autophagy-induced degradation of granzyme B released from activated NK cells in the tumor microenvironment (49, 50). The hypoxia-induced onset of autophagy has been described mechanistically for a number of cancers, including renal cancer (51), glioblastoma (52), bladder cancer (53), lung cancer (54) and acute myeloid leukemia.(55) Mechanistic insights in renal cancer have implicated HIF-2α to transcriptionally upregulate inositol triphosphate receptor 1 (ITPR1), a regulator of autophagy through calcium signaling (5).

The strength of the antitumor response of NK cells is largely dependent on the expression of NKG2D ligands on NK cells, and corresponding MICA/B receptors on tumor cells (56). These receptors are highly present in tumor environments in response to cellular stresses, and the MICA/B receptors assist the NK cells in locating the cancer cells for elimination through NKG2D receptor activation. Shedding of MIC receptors—alongside CD16 shedding— has been widely described to contribute to immune evasion and deficiency of adoptive NK cell transfers (57–59). This shedding has shown to be induced by hypoxia through altered nitric oxide (NO) signaling (60), and could be rescued through exogenous induction of NO signaling (61). It bears mentioning that shedding of CD16 from NK cells also occurs in response to various activation stimuli, including cytokines, cross-linking with activating receptors or target cell stimulation, ultimately resulting in decreased intracellular cytokine production and impaired CD107a degranulation (62). Without CD16 shedding, NK cell viability is not sustained, and shedding is thought to possibly prevent activation-induced cell death and modulate NK cell effector functions through the ability to engage multiple target cells.

Because of the broad and potent role of cytokines in activation of NK cells, it is no surprise that studies have investigated the implication of cytokine stimulation in hypoxic environments. Recent reports have indicated that hypoxia-induced loss of NK cell cytotoxicity could be rescued by treatment with high doses (1,000 IU/mL) IL-2 (63) for 14–16 h. While IL-2 treatment abrogated impairment of NK-mediated cytotoxicity against K562 cells *in vitro* under both normoxic (20% oxygen) and hypoxic (0% oxygen) conditions, it is still unclear how such observations translate *in vivo* against solid tumor targets. Similarly to IL-2, IL-15 induces potent cytotoxic responses on NK cells. Interestingly, short-term hypoxia (6 h, 1% O_2_) enhanced the cytotoxic response of NK cells that were simultaneously stimulated with IL-15 (50 ng/mL) against K562 cells *in vitro*, and induced upregulation of HIF-1α-induced glycolytic gene expression (8). This did not, however, result in changes in glycolytic flux compared to non-IL-15-stimulated NK cells, showing that other factors may also be involved in this response during short-term hypoxia. The synergy between IL-15 and short-term hypoxia has also resulted in the downregulation of the glycolytic/pentose phosphate pathway-linked gene TKTL1, suggesting a possible switch to Warburg metabolism in response to IL-15 and short-term hypoxia. Transcriptional microarray data collected from NK cells under hypoxia and IL-5 activation (6 h) have also been reported (64).

## Effects of adenosinergic metabolism on NK cells

Adenosine, a purine ribonucleoside, is involved in a well-characterized network of pathways in many cell types, including immune cells. While adenosine signaling occurs both intra and extracellularly, its roles diverge. Intracellularly, adenosine is involved in energy homeostasis, nucleic acid metabolism, angiogenesis, and the methionine cycle (65–67), exerting a protective effect on cells and tissues (68). Extracellular adenosine, on the other hand, is involved in intercellular signaling. Elevated extracellular concentrations of adenosine in tumors, which can be as much as 100-fold higher than in normal tissues, are known to contribute to immune evasion (69, 70). This immune evasion manifests through a combination of reduced proliferation, inhibition of cytotoxic activity, downregulation of activating receptors, and reduced secretion of cytotoxic cytokines (71). As a result, adenosinergic signaling has emerged as a negative feedback loop that regulates local and systemic anti-tumor response (72). Much is known about adenosine signaling in cells, and with deepening knowledge of its effects on NK cells (73) as a target for adoptive immunotherapy, it has become of increasing therapeutic interest (74).

The physiologic functions of adenosine are largely mediated by four types of G-protein-coupled adenosine receptors, A_1_, A_2A_, A_2B_, and A_3_, where the A_2A_ adenosine receptor (A_2A_R) is the subtype that is most frequently expressed on immune cells (75). Extracellular accumulation of adenosine in the tumor microenvironment leads to immunosuppression, particularly through A_2A_R on infiltrating immune cells, including NK cells (76–78). Indeed, effector functions of NK cells were shown to be susceptible to A_2A_R stimulation in a number of studies. Among the suppressed immune functions due to extracellular adenosine are inhibited proliferation (79), a reduced production of cytokines from IL-2-stimulated NK cells (11), and a reduced cytotoxic effector function against cancer cells (10). A2A receptor expression on tumor-associated myeloid cells was also shown to inhibit the cytotoxic function of NK cells in primary and metastatic tumor microenvironments (80), indicating that the broad effect of the A2 receptor in tumors is not limited to self-expression on NK cells. Unlike signaling through A2A, agonism of the A1 or, to a lesser extent, the A3 receptor stimulates NK cytotoxicity (77), presumably through a mechanism that involves a decrease in intracellular cAMP (81, 82). A3 agonism was also associated with higher serum levels of IL-12, a known stimulator of NK cell cytotoxicity (82). Using a different A3 receptor agonist, iodobenzyl methylcarboxamidoadenosine, that blocks the adenosine response, inhibition of proliferation, IFN-γ production, and cytotoxicity of NK cells was shown. However, through a mechanism involving the A3 adenosine receptor, and in the presence of adenosine upon stimulation with IFN-α, IFN-γ production from NK cells increased compared to that observed in the absence of adenosine (83). More recently, targeting A2AR via both A2AR inhibition or in A2AR-deficient mice resulted in improved tumor control and inhibition of tumor progression through elimination of the A2AR-induced suppression of NK cell maturation by promoting the accumulation of highly cytotoxic CD56^dim^ NK cells (84). Ongoing pre-clinical studies are also addressing the co-inhibition of the A_2A_R receptor with the A_2B_ receptor on immune cells in conjunction with chemotherapy (85); however, the A_2B_ receptor has a lower expression on NK cells compared to myeloid-derived and dendritic cells.

## Strategies to modulate immunometabolic suppression in the tumor microenvironment

Recent evidence that metabolic functions regulate NK cell activation (Table 2) has fueled interest in the preclinical development of strategies that address metabolic reprogramming of NK cells in the context of immunotherapy. While several therapeutic strategies aim to target metabolism of immune cells, approaches aimed at specifically targeting NK cell function are still emerging.

**Table 2:** Sources of immunometabolic regulation of NK cell functions.

**Source**	**Immunometabolic regulator**	**Effect on NK cells**	**References**
NK cells	mTOR (mTORC1)—activation occurs via PI3K, PDPK1, and AKT most potently by IL-15	Production of granzyme B and perforin, enhancement of glycolysis	(26), (27)
NK cells	*Srebp*	Control of glucose metabolism through citrate-malate shuttle	(31)
T-cells, dendritic cells	IL-2	Increase in cytotoxicity of NK cells	(63)
Immune Cells	IL-15	Increase in cytotoxicity of NK cells; Upregulation of HIF-1α and downregulation of TKTL1 (during hypoxia); Glycolysis regulation through mTOR	(8)
Cancer cells/Tumor microenvironment	Adenosine	Binding to A1 or A3 receptor stimulates NK cell cytotoxicity (via a decrease in cAMP); Increase in IFNy production (with a combination of IFN-α)	(77)
Immune Cells (APCs)	IL-12	Increase in cytotoxicity of NK cells	(82)

### Cytokine stimulation

Several studies are investigating cytokine stimulation to induce activation signals and enhance *in vivo* persistence of adoptively-transferred NK cells. Among these, IL-15 has been the most utilized, due to its recognized role in NK cell development, homeostasis and activation (86). Recently, the functions of IL-15 have been linked to mammalian target of rapamycin (mTOR) (87), which is known to regulate NK cell metabolism through glycolysis. There are various clinical trials currently investigating several IL-15 dosing regimens for improving NK cell metabolism for adoptive transfer. Though IL-15-stimulated NK cells were shown to eradicate solid tumors (88), recent findings have indicated that continuous treatment with IL-15 may actually result in exhaustion of NK cells (13), ultimately leading to cells with markedly diminished cytolytic and inflammatory function. As it stands, our understanding of optimal IL-15 dosing, timing, or the overall effects of its use in the immunotherapy of adoptively-transferred NK cells is incomplete.

### Metabolic inhibition

The mTOR inhibitor rapamycin has been used in clinical immunotherapy for a number of years to suppress the metabolic activity of pathogenic cells (89). The metabolic activity of NK cells is regulated by mTOR. In NK cells, mTOR is activated primarily, though not exclusively, by IL-15 (90), rationalizing the use of IL-15 in adoptive immunotherapy. Consequently, stunting metabolic activity for cancer treatment has raised concerns that using IL-15 could lead to associated negative effects on glucose metabolism of immune cells, including NK cells (12). Recently, Yang et al. (91) described the independent roles of mTORC1 and mTORC2 in the development of NK cells: while the loss of either did not disrupt the cytolytic function of NK cells, deletion of mTORC1 function disrupted NK cell homeostasis, and the absence of mTORC2 stunted the terminal maturation of NK cells. Results like these have shown that the use of metabolic inhibitors, such as rapamycin or Torin-1, should be taken into consideration to make sure there are not any secondary and potentially suppressive effects on the immune cell effector function, since manipulation of mTOR signaling can affect NK cell development and repopulation.

### Targeting adenosinergic signaling with adoptive NK cell immunotherapy

With recent advances in immunotherapy using genetically-engineered immune cells, an emerging therapeutic approach has been to combine an A_2A_ receptor blockade with adoptive cell therapy. Targeting the A_2A_ receptor with a small molecule inhibitor, such as SCH58261, or with A_2A_R knockdown in cells using shRNA, triggers the enhancement of the cytotoxic function of anti-HER2 CAR-T cells either alone or in combination with anti-PD-1 therapy when transferred adoptively to tumor-bearing C57BL/6 mice (92). Unlike CAR-T cells, however, no such studies using adoptively-transferred NK cells exist yet.

**Figure 1:**
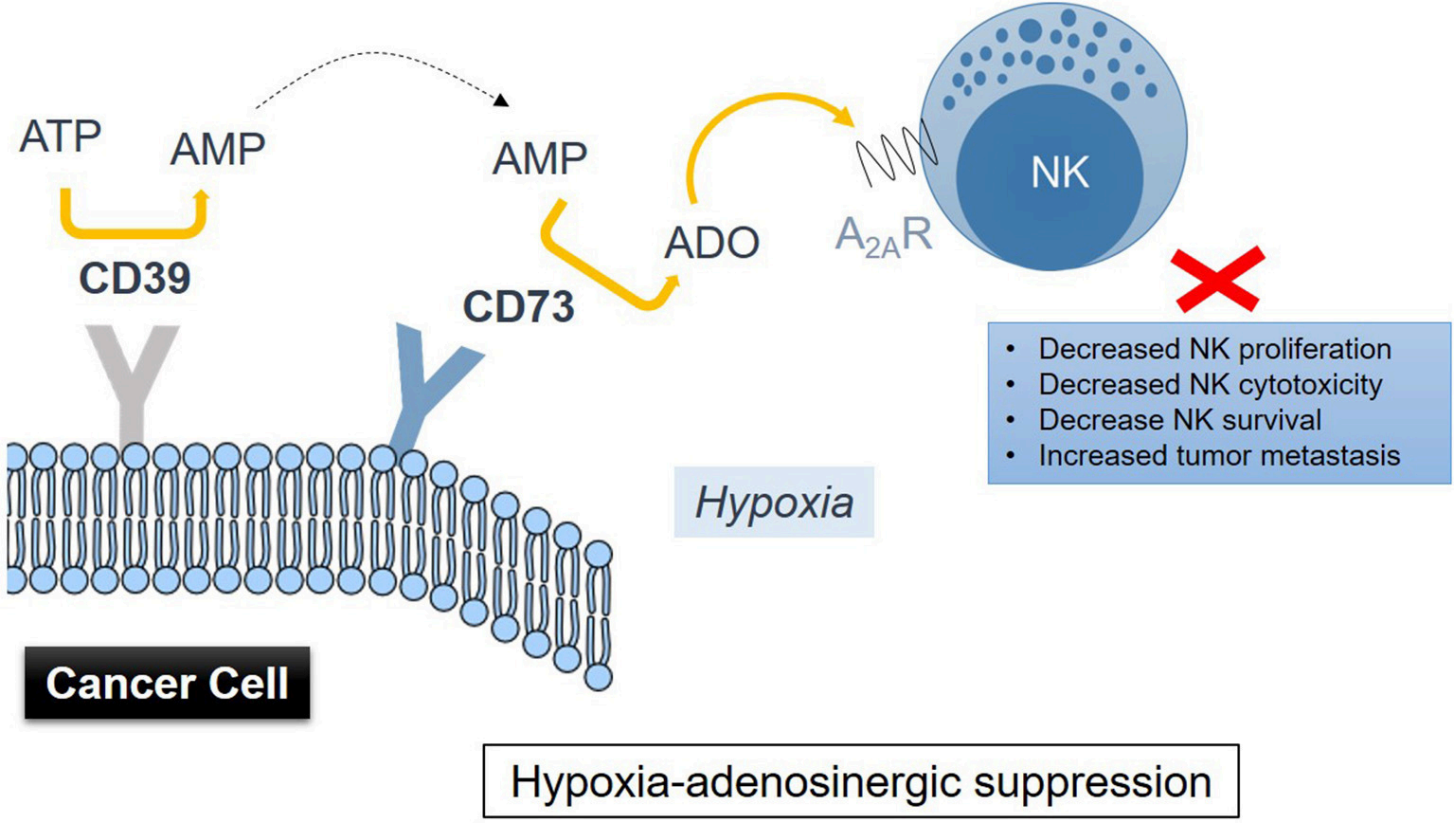
Drawing representation of immunometabolic suppression of NK cell functions induced by adenosine metabolism in the TME involving CD39 and CD73 on cancer cells via signaling through the adenosine A2A receptor on NK cells.

Accumulation of extracellular adenosine in tumors is associated with activity of the CD39-CD73 enzymatic cascade (93). Hypoxia in solid tumors promotes the release of nucleotides including AMP and ATP, which fuel the enzymatic activity of the ectonucleotidases CD39 and CD73 (Figure 1), inducing the conversion of AMP → ATP → extracellular adenosine, with CD73 catalyzing the dephosphorylation of extracellular 5′AMP to adenosine as the final step in this pathway (94). HIF-1 was implicated as regulating the activity of CD73 (95). Though CD73 is expressed on several cell types including NK cells, it is highly upregulated in various cancers (91, 96). Antibody therapy with anti-CD73 antibodies or CD73 shRNA (97, 98) was shown to be effective in inhibiting tumor growth and metastasis. This type of treatment has recently found its way into the clinic and is known as Medimmune's Oleclumab (anti-CD73 antibody), which is currently in Phase I clinical trials (99). Therapeutic inhibition of CD73 was also shown to improve antitumor efficacy of anti-PD-1 and anti-CTLA-4 checkpoint inhibitors in preclinical models of various solid tumors (100). A limited expression of CD73 is observed on NK cells, typically in the region of 1%. NK cells were reported to be able to enhance their expression of CD73 upon co-culture with mesenchymal stem cells (MSCs) (101). CD73 expression conferred by MSCs results in catalytically active ectoenzyme, and NK cells that are capable of producing higher amounts of adenosine via the conversion of 5′-AMP. Further studies have implicated the CD56^bright^CD16^−^ NK subset as most active in producing adenosine via involvement of CD38—by inhibiting CD38 activity, adenosine production was reduced (102).

Mechanisms that guide CD73-induced promotion of tumor growth and immune resistance include not only the inhibition of NK cell cytotoxicity, but also induction of the internalization of CD73 expressed on cancer cells (103). For that reason, a number of anti-CD73 clones have been indicated to promote internalization of CD73 with limited effect on its enzymatic activity. While CD73-targeting antibody therapy can potentiate the anti-tumor activity of NK cells via ADCC, a recent study showed that blockade of CD73 with anti-CD73 clone 7G2 in conjunction with anti-CD39 clone A1 enhanced NK cell cytotoxicity *in vitro* via a mechanism that includes adenosinergic metabolism and is independent of ADCC (104). Our own work showed that a CD73 antibody blockade enhances the killing potential of CAR-engineered NK-92 cells, a widely-used NK cell line that does not express CD16, via mechanisms that implicate extracellular adenosine in the absence of ADCC. NK cells were also implicated in the metastatic control of LWT1 melanoma tumors *in vivo* when C57BL/6 mice were targeted by anti-CD73 antibody therapy in conjunction with inhibition of A_2A_R signaling (105). Alongside NK cells, tumor control was optimal when CD8^+^ T cells, interferon-γ and perforin were also present. Most of the *in vivo* studies targeting CD73 use mouse clones of these antibodies and are performed in C57BL/6 mice. Therefore, further insights are needed on understanding the effect of targeting the CD73/adenosinergic pathway with human-directed antibody clones in association with adoptive transfer of immune cells into *in vivo* models that bear human immune components.

Adenosine signaling was also studied in NK-92 cells Hong et al. (106) observed that upon exposure to acute myeloid leukemia-derived exosomes, NK-92 cells, which also express the A_2A_R receptor, increased their expression of adenosine, inosine and hypoxanthine, ultimately contributing to autocrine inhibition of NK-92 cytotoxic function via upregulation of A_2A_R function (106).

Adenosine is metabolically unstable, becoming rapidly converted to inosine via the activity of the enzyme adenosine deaminase (ADA). For that reason, most studies on the effect of adenosine on NK cells use its metabolically stable analog, 2-chloroadenosine, thereby bypassing catalytic removal of rapidly-accumulating extracellular adenosine. Extreme dysfunction in purinergic metabolism can lead to adenosine deaminase-severe combined immunodeficiency (ADA-SCID), which causes the accumulation of adenosine due to ADA loss of function, ultimately resulting in partial or complete lymphopenia (107).

Though direct studies on the effects of purine metabolism, particularly that of adenosine, on the functions and phenotypes of NK cells are limited, insights are starting to emerge. McCarthy et al. (108) observed that the generation of purine nucleotides, including adenosine, through glycolysis drives the expression of MICA ligands on cells, which are then targeted by NKG2D receptors on NK cells. Restricting early glycolytic pathway intermediates abrogated the expression of MICA, showing that cell proliferation was not shown to be a prerequisite for MICA expression.

## Conclusion

An emerging body of work is starting to highlight the critical role of immunometabolism in innate immunity and the development of emerging cancer immunotherapies. Among the immunosuppressive features of tumors is hypoxia, which results in the generation of adenosine, a metabolite that is highly suppressive to NK cell cytotoxicity and proliferation. These immunometabolic changes are specific to NK cells and occur under various cytokine stimulation programs. While mTOR has been recognized as a key driver of metabolic reprogramming in NK cells, the mechanisms by which mTOR regulates the metabolic system and NK cell effector responses in the tumor microenvironment are still largely unknown. Moreover, the role of mTOR-mediated regulation of protein translation during NK cell effector responses has not been studied in depth. Limited knowledge also exists on how these metabolic changes occur in phenotypically distinct NK subsets, particularly licensed cells. For instance, metabolic responses of licensed and unlicensed NK cells are not known, and neither are the metabolic programs of NK cells that display exhausted functional phenotypes. This dearth of knowledge precludes our utilization of NK cells as effective adoptive NK immunotherapies; however, rapid advances are fueling remarkable discoveries in the field. Future immunotherapies with adoptively-transferred NK cells are expected to employ modalities that reverse or avoid immunometabolic suppression of NK cell function in their design to ultimately lead to the improved targeting of solid tumors.

## Author contributions

SM researched the literature and wrote the initial draft of the manuscript. AC and KL performed the literature review, generated the tables and prepared an edited version of the manuscript. AC, KL, and SM critically revised the manuscript. AC, KL, and SM wrote the manuscript and edited the figure and tables.

### Conflict of interest statement

The authors declare that the research was conducted in the absence of any commercial or financial relationships that could be construed as a potential conflict of interest.
